# Overexpression of RAD54L attenuates osteoarthritis by suppressing the HIF-1α/VEGF signaling pathway: Bioinformatics analysis and experimental validation

**DOI:** 10.1371/journal.pone.0298575

**Published:** 2024-04-09

**Authors:** Zhengnan Li, Lifeng Xie, Longqiang Zou, Shiliang Xiao, Jun Tao

**Affiliations:** 1 Department of Orthopedics, The Second Affiliated Hospital of Nanchang University, Donghu District, Nanchang City, Jiangxi Province, China; 2 Department of Sports Medicine, The Affiliated Ganzhou Hospital of Nanchang University (Ganzhou People’s Hospital), Zhanggong District, Ganzhou City, Jiangxi Province, China; Guangzhou University of Chinese Medicine, CHINA

## Abstract

Osteoarthritis (OA) is a widespread chronic, progressive, degenerative joint disease that causes pain and disability. Current treatments for OA have limited effectiveness and new biomarkers need to be identified. Bioinformatics analysis was conducted to explore differentially expressed genes and DNA repair/recombination protein 54 L (RAD54L) was selected. We firstly overexpressed RAD54L in interleukin-1β (IL-1β)-induced human articular chondrocytes or in OA rats to investigate its effect on OA. Chondrocyte viability and apoptotic rate were measured by Cell Counting Kit-8 and flow cytometry, respectively. Then we evaluated OA severity *in vivo* by Hematoxylin-eosin staining and Osteoarthritis Research Society International standards. The expression of inflammatory mediators was tested by enzyme-linked immunosorbent assay. Finally, western blot was performed to determine the relative expression level of hypoxia-inducible factors 1α (HIF-1α) and vascular endothelial growth factor (VEGF). Overexpression of RAD54L promoted cell viability and attenuated apoptosis in IL-1β-induced human chondrocytes. A lower Osteoarthritis Research Society International score and a remarkable alleviation of chondrocyte disordering and infiltration of inflammatory cells were found in cartilage tissues of OA rats after overexpressing RAD54L. The inflammatory response induced by OA was decreased by RAD54L overexpression *in vitro* and *in vivo*. In addition, RAD54L overexpression decreased the relative expression level of HIF-1α and VEGF. Overexpression of RAD54L could attenuate OA by suppressing the HIF-1α/VEGF signaling pathway, indicating that RAD54L may be a potential treatment target for OA.

## Introduction

As a prevalent whole-joint degenerative disease, involving all joint tissue including cartilage, infrapatellar fat pad, meniscus, synovial membrane and subchondral bone, osteoarthritis (OA) has affected the lives of more than 500 million people worldwide [1, 2]. Personal factors, including age, gender, obesity, genetics, and diet, and joint-level factors, such as injury and malalignment, are known risk factors for OA [3, 4]. Furthermore, OA is a leading cause of disability, resulting in a financial burden for both patients and healthcare systems [1, 5, 6]. However, there is still no certain cure for OA. Surgical therapies [7] and non-pharmacological approaches like lifestyle modification [8] are considered as the common strategies for the treatment of OA [9]. Traditional medical treatments help relieve symptoms, but are often accompanied by obvious side effects [10, 11]. Hence, it is imperative to unravel the molecular pathogenic mechanisms and identify novel potential targets in patients with OA.

The DNA repair/recombination protein 54 L (RAD54L), which was identified using bioinformatics analysis in this study, is an indispensable member belonging to the DNA-dependent ATPase Switch 2/Sucrose non-fermentable 2 protein family [12, 13]. An increasing number of studies based on public databases have revealed that RAD54L has the potential to become a new therapeutic target and an early diagnostic biomarker for cancer. For example, RAD54L expression levels are significantly correlated with different stages of non-small-cell lung cancer [14]. RAD54L together with other novel susceptibility genes have been shown the highest frequency in familial nasopharyngeal carcinoma [15]. In addition, the cell division cycle 7-RAD54L pathway is functionally required for tumorigenicity and radio resistance of glioblastoma [16]. Bladder cancer patients with a high expression of RAD54L have a relatively shorter survival time [17]. However, few studies have investigated the role of RAD54L in the progression of OA.

Hypoxia-inducible factor 1α (HIF-1α) is a key member of the hypoxia-inducible factor family, members of which regulate the transcription of genes involved in angiogenesis, inflammation [18], autophagy [19], and apoptosis [20], and take part in the progression of OA [21]. Vascular endothelial growth factor (VEGF) has been reported to be involved in increasing vascular permeability [22] and regulating cell proliferation, migration, and apoptosis [23–25]. In recent years, the HIF-1α/VEGF signaling pathway has been found to take part in the progression of OA. For example, in OA of the temporomandibular joint, hypoxia promotes the angiogenesis of condylar cartilage by activating the HIF-1-VEGF-Notch signaling pathway [26]. Resveratrol can alleviate pain, ameliorate swollen joints, and reduce inflammation markers via signal transducer and activator of transcription 3/HIF-1/VEGF signaling pathway, showing protective effects on OA [27]. Whether RAD54L can regulate the HIF-1α/VEGF pathway in OA is still unknown.

Here, bioinformatic analysis was conducted to explore differentially expressed genes (DEGs), and RAD54L was identified. We explored the potential effects of RAD54L on OA in vivo and in vitro and found that overexpression of RAD54L attenuated OA by suppressing the HIF-1α/VEGF signaling pathway. Our results may offer more insights into exploring the clinical diagnosis and treatment of OA.

## Materials and methods

### Microarray data source

Gene expression profiling in this study was downloaded from the Gene Expression Omnibus (GEO) database (https://www.ncbi.nlm.nih.gov/geo/). Taking “osteoarthritis” as a keyword, two datasets, including GSE98918 and GSE51588 were selected. The GSE98918 dataset was based on the GPL20844 platform (Agilent, Santa Clara, CA, USA) and the data are from meniscal tissues obtained from 12 patients with OA or not. Similarly, the GSE51588 dataset analysis was based on the GPL13497 platform (Agilent) and has data from subchondral bone samples from 12 OA and 5 non-OA patients.

### Identification of DEGs

The DEGs between OA and normal tissue samples (Control) were identified by GEO2R with |logFC| ≥1 and an adjusted P-value ≤ 0.05 as the thresholds for DEGs. Volcano plots were used to visualize the DEGs. Data correction and standardization were achieved through the boxplots. A specific DEG was defined as a common DEG (co-DEG) when it was present in both the meniscal tissues and subchondral bone tissues. Co-DEGs between GSE98918 and GSE51588 were obtained using the online tool EVenn (http://www.ehbio.com/test/venn/#/), based on the expression levels of the co-DEGs in GSE98918 and GSE51588. Heatmaps were constructed for visualization by https://www.bioinformatics.com.cn [28].

### Functional enrichment analysis of the DEGs

Gene ontology (GO) and Kyoto Encyclopedia of Gene and Genome (KEGG) functional pathway analysis was conducted using the Database for Annotation, Visualization, and Integrated Discovery (DAVID, https://david.ncifcrf.gov/summary.jsp). We assessed the results through R software with a minimum P-value considered the most significant.

### Protein-protein interaction (PPI) network construction and identification of hub genes

The PPI network was created using the Search Tool for the Retrieval of Interacting Genes Database (STRING, https://www.string-db.org/) for interacting protein-coding genes involved in the pathogenesis of osteoarthritis, based on a confidence score of 0.15. Cytoscape software (www.cytoscape.org/) was used to visualize the images, and the critical interacted genes were screened by the Molecular Complex Detection plug-in (degree cutoff = 2, node score cutoff = 0.2, K-core = 2, and max depth = 100).

### Hub gene analysis

Firstly, we made a boxplot to show the expression difference between the Control group and the OA group with the help of the expression data of hub genes in GSE98918. Then the expression ridgeline plot was drawn to show the distribution of genes in different sample data. A chordal graph was performed to reveal the expression changes of hub genes involved in the GO terms by https://www.bioinformatics.com.cn [28]. The potential diagnostic value of each hub gene was assessed using the receiver operating characteristic (ROC) curve through the website (http://gepia.cancer-pku.cn/) and the area under the curve. Ridge mapping and principal component analysis of the hub genes were performed.

### Construction of a rat model of OA

Lentiviruses harboring lentiviral vectors RAD54L (Lv-RAD54L) and empty vectors (Lv-NC) were generated by GeneChem Co., Ltd. (Shanghai, China) and 6-8-week-old male Sprague-Dawley rats weighing 190–210 g were purchased from SPF (Beijing) Biotechnology Co., Ltd. (Beijing, China). Briefly, lentiviral vectors were transfected in 293T cells (Saibaikang Biotechnology Co., Ltd., Shanghai China) in Dulbecco’s modified Eagle medium (DMEM; Gibco, Grand Island, NY, USA), which was replaced with fresh complete medium after 8 h. We then collected the supernatant after 48 h of culturing and obtained the lentiviral particles after ultracentrifugation at 50,000 × g for 70 min at 4°C [29] followed by the determination of the viral titer.

The animals were randomly divided into four groups: Control, OA, Lv-NC, and Lv-RAD54L. Each group contained 6 rats, which were housed in an environment controlled at 25°C under the light/dark cycle of 12/12 h. We mixed a 5% papain solution with 0.03 mol/L L-cysteine at a 1:1 ratio and left the mixture to stand for 30 min. On days 1, 4, and 7, 0.2 mL of the mixed solution was injected vertically into the right knee joint cavity of rats in the OA, Lv-NC, and Lv-RAD54L groups [29]. Rats in the Control group were injected with the same amount of normal saline. Lentiviruses expressing RAD54L (1×10^9^ pfu in 10 μL) or empty vector lentiviruses were injected into the intra-articular joint cavity using a microsyringe once per week until the rats were sacrificed [29, 30] to overexpress RAD54L (Lv-RAD54L group) or perform as a negative control (Lv-NC group). To monitor OA symptoms, the joint swelling of rats’ knees was assessed every three days. Six weeks after the last injection, an intraperitoneal injection of 1% sodium pentobarbital (45 mg/kg) was administered to anesthetize rats for blood collection and then cervical dislocation was conducted to kill the rats for collection of the knee articular cartilage. All animal experimental procedures were performed with the approval of the Ethics Committee of Nanchang University (Approval no.: SYXK(Gan)-2021-0004).

### Immunohistochemical staining

The expression level of cleaved-caspase-3 was measured using immunohistochemical staining. Articular cartilage tissues were embedded in paraffin after fixing in 4% paraformaldehyde and were then cut into 8 μm sections. The sections were incubated with a primary antibody rabbit anti-cleaved-caspase‑3 at 37°C overnight. Then the anti-rabbit IgG (ab150077, 1:200, Abcam, Cambridge, UK) was applied to the sections, which were washed 3 times with phosphate-buffered saline. To visualize cleaved-caspase-3, the sections were stained with 3.3-diaminobenzidine and then observed by light microscopy.

### Histopathologic analysis

Each cartilage sample embedded in paraffin was cut into 4 μm sections and stained with hematoxylin and eosin (H&E) to evaluate the morphological changes of the knee articular cartilage tissues. The sections were analyzed under a microscope (Eclipse 80i, Nikon, Tokyo, Japan). The level of inflammation was evaluated using an inflammation scoring system based on the H&E staining results [31]. We used the OA Research Society International (OARSI) scoring system to evaluate the pathological changes in OA cartilage tissues. Each section was scored in a blinded fashion by two individuals [32].

### Cell culture, treatment, and transfection

Human primary articular chondrocytes (Saibaikang Biotechnology Co.) were cultured in DMEM (Gibco) containing 10% fetal bovine serum (Gibco) and 1% streptomycin/penicillin antibiotics, and maintained in a humidified incubator under 5% CO_2_ at 37°C. Interleukin-1β (IL-1β) is an important pro-inflammatory cytokine that can induce chondrocyte apoptosis, leading to cartilage matrix degradation and joint inflammation, and thus, promoting the progression of OA [33, 34]. Therefore, the chondrocytes were exposed to 10 ng/mL IL-1β (Med Chem Express, Monmouth Junction, NJ, USA) for 24 h to construct the OA cell model [35].

The RAD54L-overexpression plasmid (pcDNA3.1-RAD54L) was acquired from Shenggong Bioengineering Company (Shanghai, China). Cells induced with IL-1β were transfected with pcDNA3.1-RAD54L or the empty plasmid, pcDNA3.1-NC, using Lipofectamine 3000 (Life Technologies Corporation, Carlsbad, CA, USA) for 48 h to overexpress RAD54L (IL-1β+oe-RAD54L group) or as a control (IL-1β+oe-NC group), respectively. Therefore, the cells were divided into 4 groups: Control (chondrocytes without any treatment), IL-1β, IL-1β+oe-NC, and IL-1β+oe-RAD54L groups.

### Real-time quantitative polymerase chain reaction (RT-qPCR)

Total RNA was extracted from chondrocytes or articular cartilage tissues using TRIzol reagent (Invitrogen Life Technologies, Carlsbad, CA, United States). A 2 μL sample was taken to determine the concentration. The RNA was reverse transcribed into cDNA using the PrimeScript RT Reagent kit (Vazyme, Nanjing, China). RT-qPCR was performed on ABI7500 FAST Real-Time PCR (Applied Biosystems, Foster City, CA, USA) by the SYBR Green master mix with GAPDH as the housekeeping gene. The cycling conditions were an initial denaturation at 95°C for 30 s, followed by 40 amplification cycles of 95°C for 10 s and 60°C for 30 s. The detailed primer information was shown in S1 Table.

### Cell Counting Kit-8 (CCK-8)

Transfected cells at a density of 2 × 10^4^ cells /well were transferred to 96-well plates and cultured for 24 h. Then 10 μL CCK-8 solution (Solarbio, Beijing, China) was added to each well and the cells were incubated for an additional 2 h in the dark conditions. The optical density (OD) was evaluated using a full-wavelength microplate analyzer at an absorbance wavelength of 450 nm.

### Enzyme-linked immunosorbent assay (ELISA)

The levels of IL-18, IL-6, and tumor necrosis factor-α (TNF-α) in the cell supernatant and the serum of rats were measured with an ELISA kit (Esebio, Shanghai, China) based on the manufacturer’s protocol. The OD values were recorded under a microplate reader at a wavelength of 450 nm.

### Flow cytometry assay

For apoptosis analysis, cells were stained with a fluorescein isothiocyanate (FITC)-conjugated anti-annexin V antibody and propidium iodide (Solarbio). Briefly, the cells at a density of 1×10^6^ cells/well were inoculated into a six-well plate and incubated at 37°C for 48 h. The harvested cells were treated with 5 μL of annexin V-FITC and 5 μL of propidium iodide in the dark for 15 min at room temperature. The fluorescence intensity was detected using a flow cytometer and quantified using FlowJo software (Becton Dickinson, Ashland, OR, USA).

### Western Blot assay

We detected the expression levels of RAD54L, HIF-1α, and VEGF with the help of Western Blot assay. Proteins extracted from chondrocytes or cartilage tissues were isolated using a 10% sodium dodecyl sulfate-polyacrylamide gel electrophoresis and transferred onto a polyvinylidene fluoride membrane (Millipore, Danvers, MA, USA). The membrane blocked by 5% skim milk was exposed to primary antibodies which included anti-RAD54L (A20181, 1:1000, ABclonal, Wuhan, China), anti-HIF-1α (ab179483, 1:1000, Abcam), anti-VEGF (A5708, 1:1000, ABclonal), and anti-GAPDH (ab181602, 1:10000, Abcam) antibodies at 4°C overnight. The membrane was then incubated with a horseradish peroxidase-conjugated anti-rabbit secondary antibody (ab288151, 1:5000, Abcam) at room temperature for 1 h. The proteins after exposure were visualized using the Tanon 5200 chemiluminescence imaging system (Tanon, Shanghai, China).

### Statistical analysis

Statistical analyses were performed using GraphPad Prism 8.0 (GraphPad, San Diego, CA, USA). Quantitative data are presented as means ± standard deviation. Student’s t-test was used to compare differences between two groups, and differences among multiple groups were determined by one-way ANOVA with Tukey’s post hoc analysis. All experiments were performed at least in triplicate. P < 0.05 was considered a statistically significant difference.

## Results

### Identification of DEGs

**Fig 1:**
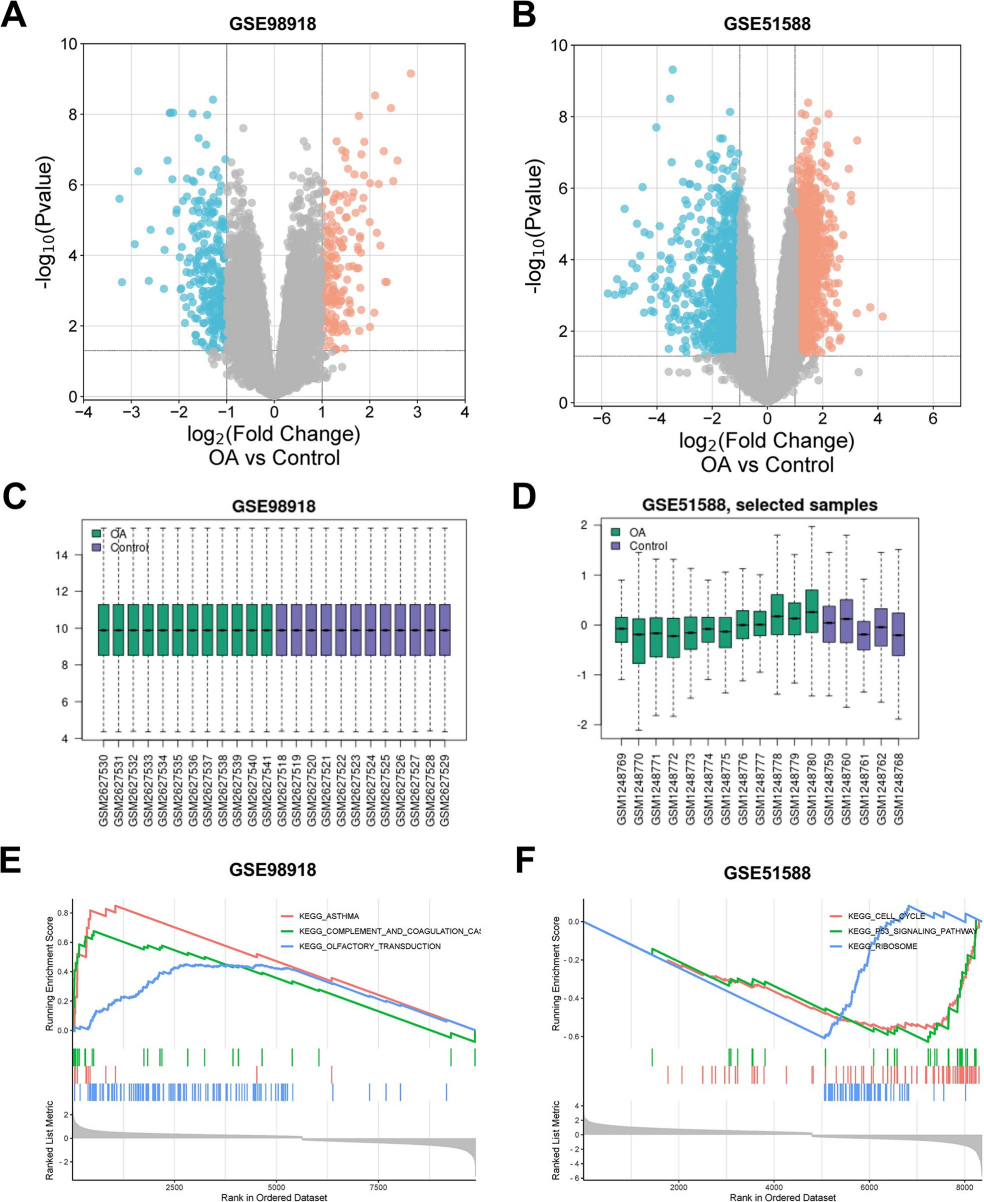
Identification of differentially expressed genes (DEGs). **(A)** and **(B)** The volcano maps of DEGs in GSE98918 and GSE51588 respectively; red points presented up-regulated DEGs, and blue points presented down-regulated DEGs. **(C)** and **(D)** The box line plots showed the data correction results of the selected samples in GSE98918 and GSE51588 respectively. **(E)** and **(F)** The top 3 pathways of gene set enrichment analysis of DEGs in GSE98918 and GSE51588 respectively.

The GSE98918 and GSE51588 datasets were obtained from the GEO database. We then selected 12 normal meniscus tissue samples (Control) and 12 OA meniscus tissue samples from the GSE98918 dataset and obtained 502 DEGs based on a threshold of |logFC| ≥ 1 and an adjusted P-value ≤ 0.05, including 191 up-regulated genes and 311 down-regulated genes. A total of 17 samples (5 normal subchondral bone samples and 12 OA subchondral bone samples) from GSE51588 were selected and 2299 DEGs were detected which consisted of 1387 up-regulated genes and 912 down-regulated genes, which were visualized using volcano plots after cluster analysis (Fig 1A and 1B). Data correction and standardization of the GSE98918 and GSE51588 datasets were achieved through the boxplots (Fig 1C and 1D). The top 3 pathways identified in gene set enrichment analysis of DEGs in each dataset were shown in Fig 1E and 1F.

**Fig 2:**
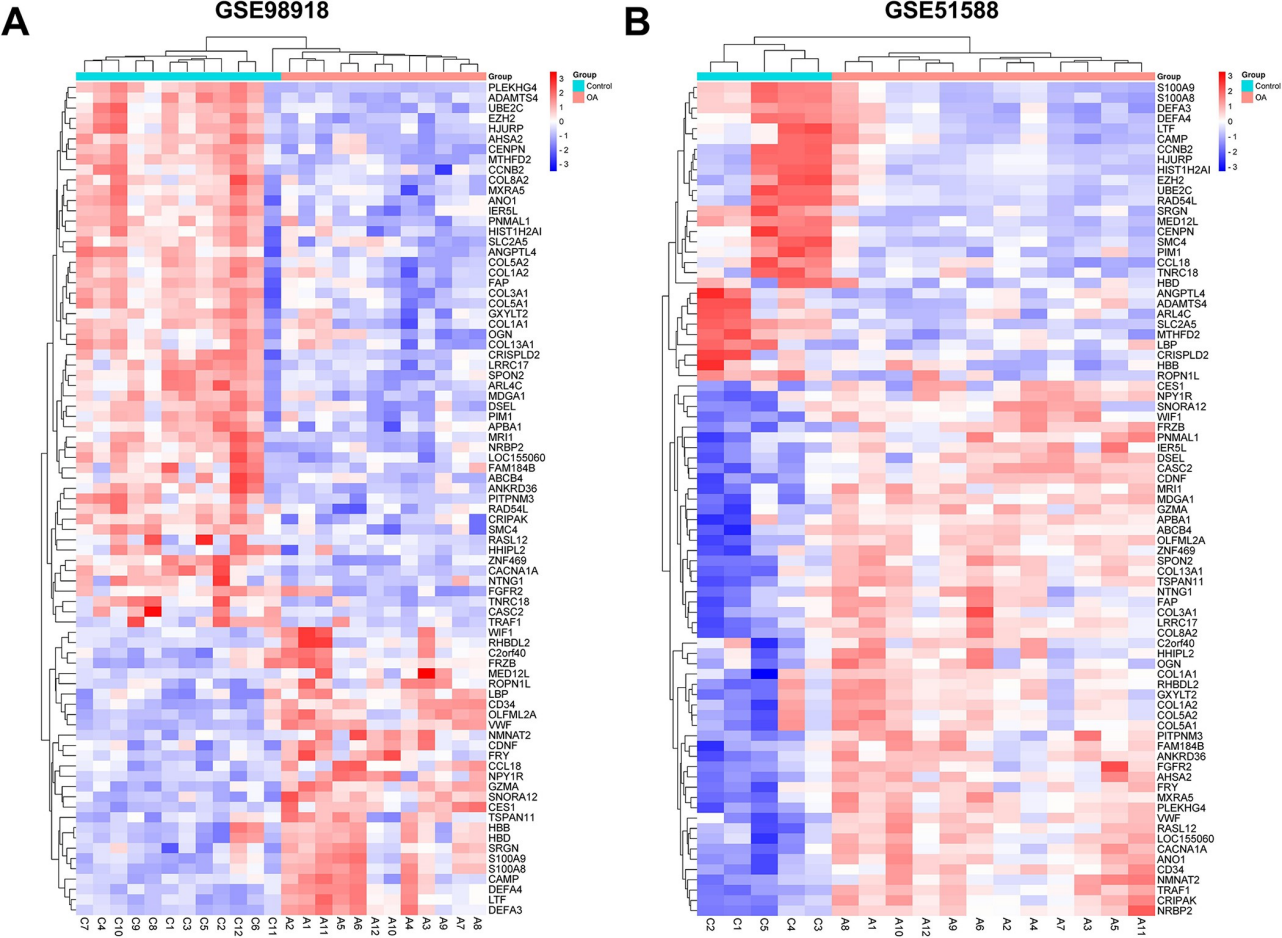
Heatmaps of common DEGs (co-DEGs). **(A)** and **(B)** The heatmaps of co-DEGs in GSE98918 and GSE51588 respectively. Reprinted from [28] under a CC BY license, with permission from https://www.bioinformatics.com.cn, original copyright 2023.

There were 81 co-DEGs between the two datasets, as shown in the Venn diagram in S1 Fig. Heatmaps of the expression levels of co-DEGs in the GSE98918 and GSE51588 datasets were displayed in Fig 2A and 2B, respectively.

### GO and KEGG analysis

**Fig 3:**
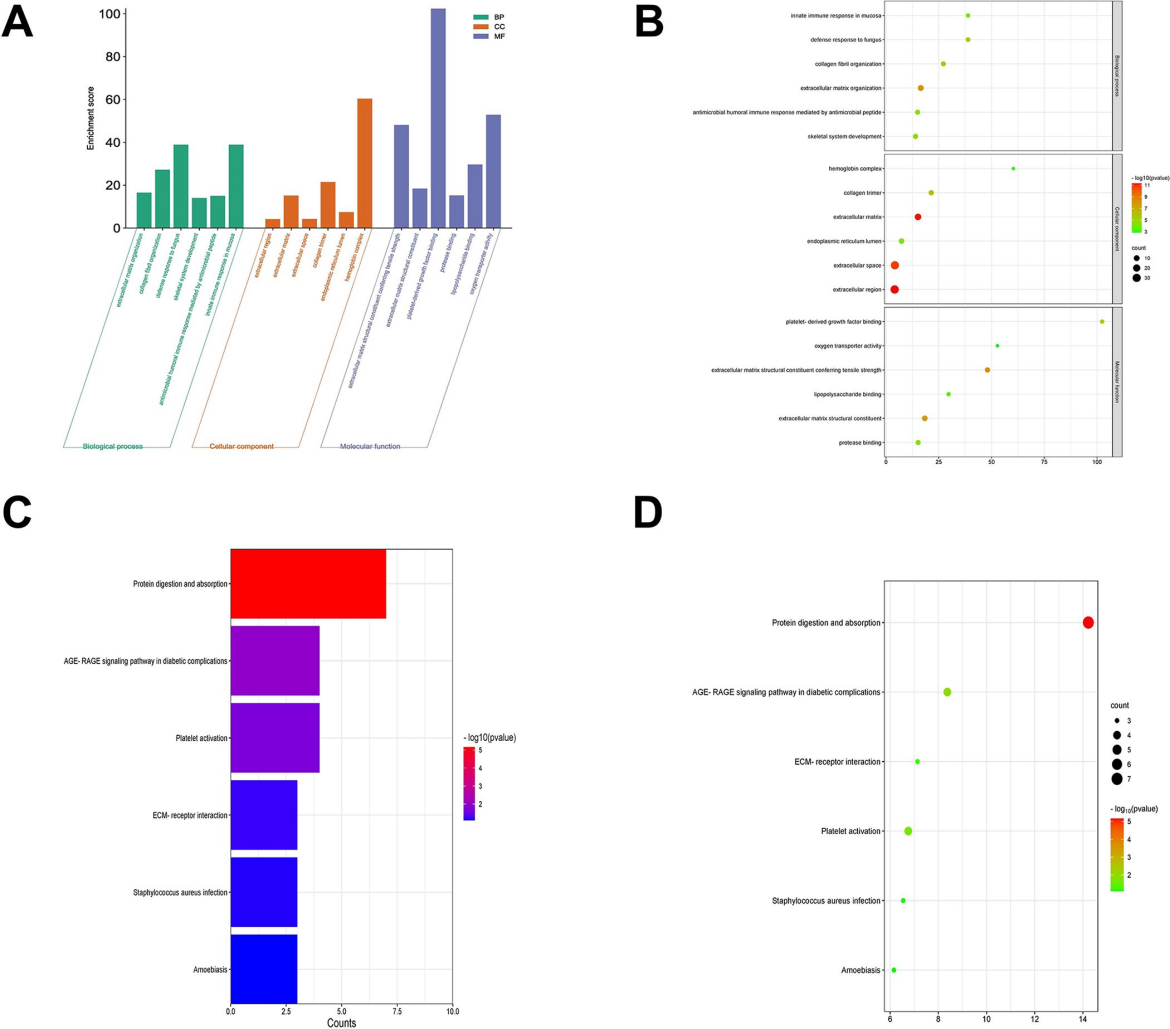
Functional enrichment analysis of co-DEGs. **(A)** A histogram and **(B)** A bubble diagram of Gene Ontology enrichment analysis of co-DEGs. **(C)** A histogram and **(D)** A bubble diagram of the Kyoto Encyclopedia of Genes and Genomes pathway analysis co-DEGs.

GO and KEGG analysis of the 81 co-DEGs were obtained using DAVID. The GO enrichment analysis for the biological process revealed that co-DEGs were mainly enriched in innate immune response in mucosa, defense response to fungus, and antimicrobial humoral immune response mediated by antimicrobial peptide. For the molecular function, these co-DEGs were primarily enriched in platelet-derived growth factor binding and oxygen transporter activity. For the cellular component, these co-DEGs were notably enriched in extracellular matrix, collagen trimer, endoplasmic reticulum lumen and hemoglobin complex (Fig 3A and 3B). Additionally, the co-DEGs were mainly involved in the Advanced Glycation End Product (AGE)-Receptor for AGE (RAGE) signaling pathway in diabetic complications, platelet activation and the extracellular matrix (ECM)-receptor interaction signaling pathway (Fig 3C and 3D).

### PPI network construction

A PPI network based on co-DEGs was constructed using the STRING database and visualized using Cytoscape software (S2A Fig). The 3 most densely connected modules of the PPI network were shown in S2B Fig. Based on our results and those from the literature, we selected RAD54L, holliday junction recognition protein (HJURP), ubiquitin-conjugating enzyme E2C (UBE2C), cyclin B2 (CCNB2), structural maintenance of chromosome 4 (SMC4), and centromere protein N (CENPN) in module 2 as the hub genes.

### Hub gene analysis

**Fig 4:**
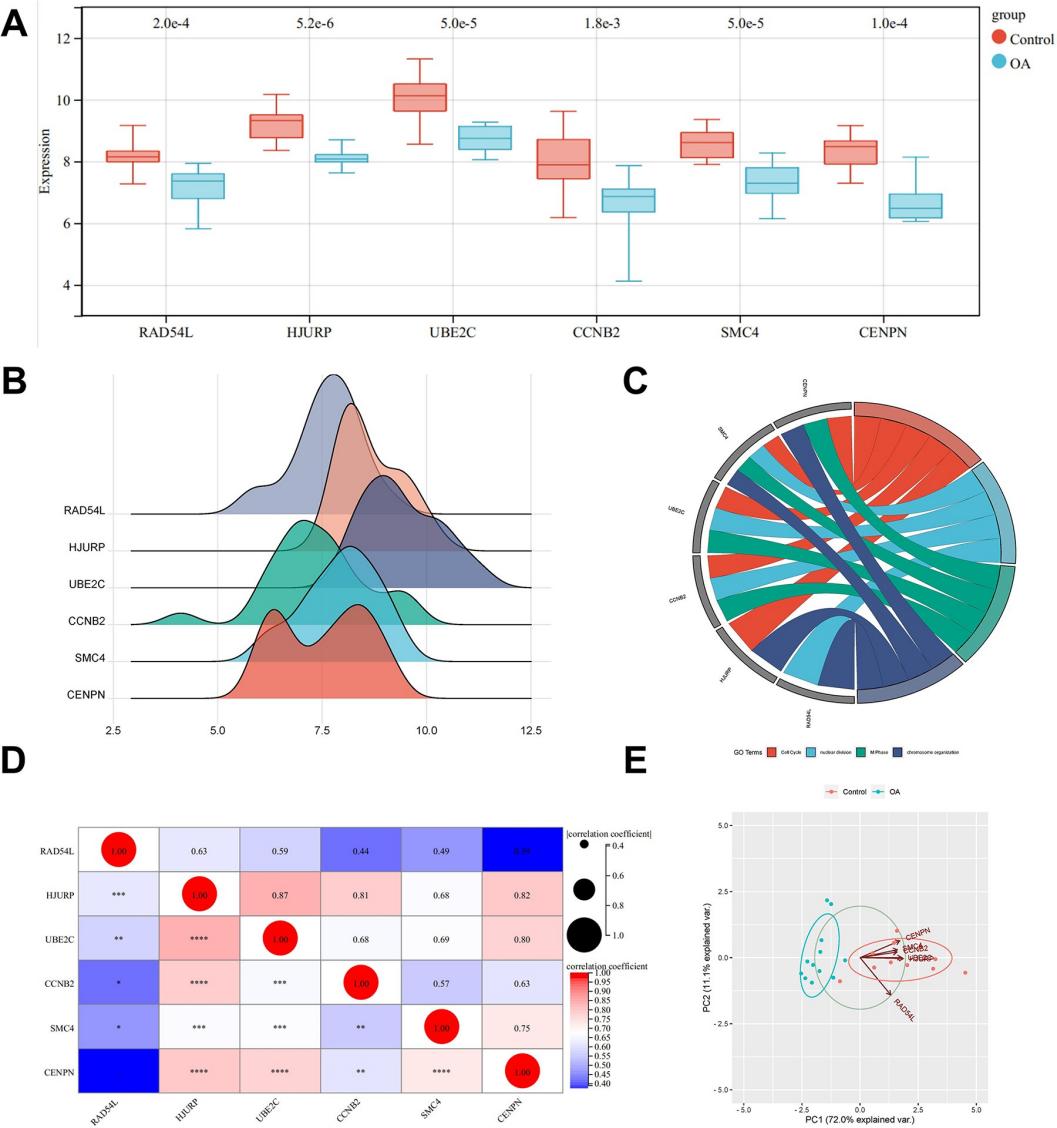
Hub gene analysis. **(A)** A box line plot showed the expression difference between the Control group and the OA group in GSE98918. **(B)** A ridge diagram of hub genes. The horizontal axis stands for the gene expression, the shape of the peak stands for the dispersion among data, and the height stands for the number of samples corresponding to the gene expression. **(C)** A chordal graph with the expression changes of hub genes involved in the GO terms. Reprinted from [28] under a CC BY license, with permission from https://www.bioinformatics.com.cn, original copyright 2023. **(D)** Matrix correlation analysis chart. **(E)** Principal component analysis of hub genes.

A boxplot was drawn to visualize the difference in gene expression levels between the Control group and the OA group. The results showed that the expression levels of hub genes were decreased in OA tissues (Fig 4A). A ridge diagram was constructed to verify the reliability of these six hub genes (Fig 4B). We obtained a chordal graph to show the correlation between the hub genes and the GO terms (Fig 4C) and constructed a matrix analysis diagram to reveal the internal correlation between hub gene expression levels in GSE98918 and GSE51588 (Fig 4D). Principal component analysis was performed using the expression levels of the six hub genes in the GSE98918 dataset as variables, and the results were processed by R software package. The scatter plots showed that the two principal components (PC1 and PC2) effectively explained 83.1% of the difference between the Control group and the model group, and taking them as the horizontal and vertical coordinates in the scatter plot, two groups of samples could be separated well (Fig 4E).

### Validation of diagnostic values of hub genes

Receiver operating characteristic curves showed that using RAD54L, HJURP, UBE2C, CCNB2, SMC4, or CENPN as indicators, the true positive rates were 91.7%, 97.9%, 94.4%, 86.1%, 94.4% and 93.1% respectively in the GSE98918 dataset (S3A Fig), and 66.7%, 63.3%, 78.3%, 60%, 95% and 100% respectively in the GSE51588 dataset (S3B Fig). Therefore, these hub genes have effective diagnostic value in distinguishing OA from control samples, indicating that they may be considered as potential biomarkers for OA.

### Expression analysis of hub genes in OA model rats

**Fig 5:**
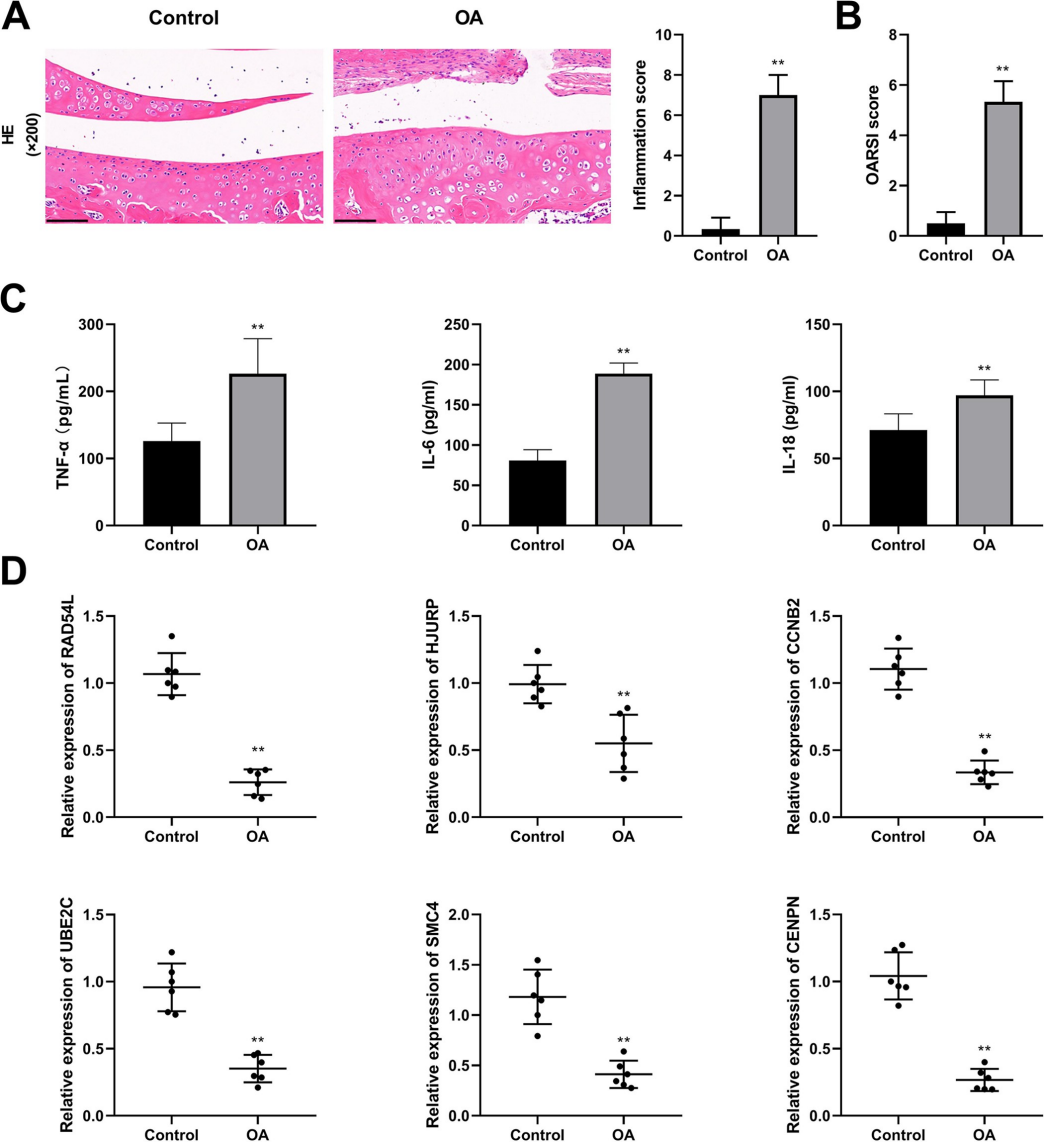
The expression of hub genes in osteoarthritis (OA) model rats. **(A)** The results of hematoxylin-eosin staining (200×, scale bars = 100 μm) and the inflammation score. **(B)** The Osteoarthritis Research Society International score. **(C)** The levels of proinflammatory cytokines (TNF-α, IL-6, and IL-18) in serum. **(D)** The relative mRNA expression of RAD54L, HJURP, UBE2C, CCNB2, SMC4, and CENPN. ** P < 0.01 *vs*. the Control group.

To further verify the differences in expression of the six hub genes, we constructed OA model rats. The results of H&E staining revealed that the growth plates of the cartilage tissues in the Control group presented a regular arrangement and there were no inflammatory cells. Compared with the Control group, the number of normal cells in the OA group was greatly reduced, the number of inflammatory cells was significantly increased, and the cells were in an irregular arrangement (Fig 5A). Therefore, a higher inflammation score and a higher OARSI score were observed in the OA group (P < 0.01, Fig 5A and 5B). The serum levels of pro-inflammatory cytokines (IL-18, IL-6, and TNF-α) were significantly higher in rats in the OA group than those in the control group (P < 0.01, Fig 5C). These results demonstrated that the proposed modeling method was feasible and successful. Subsequently, we confirmed the mRNA expression levels of hub genes in the cartilage tissues and found that all of them were down-regulated in the OA model (P < 0.01, Fig 5D), which was consistent with the results of the bioinformatics analysis.

### RAD54L overexpression promotes cell viability and inhibits the inflammatory response and the apoptosis of IL-1β-induced human chondrocytes

**Fig 6:**
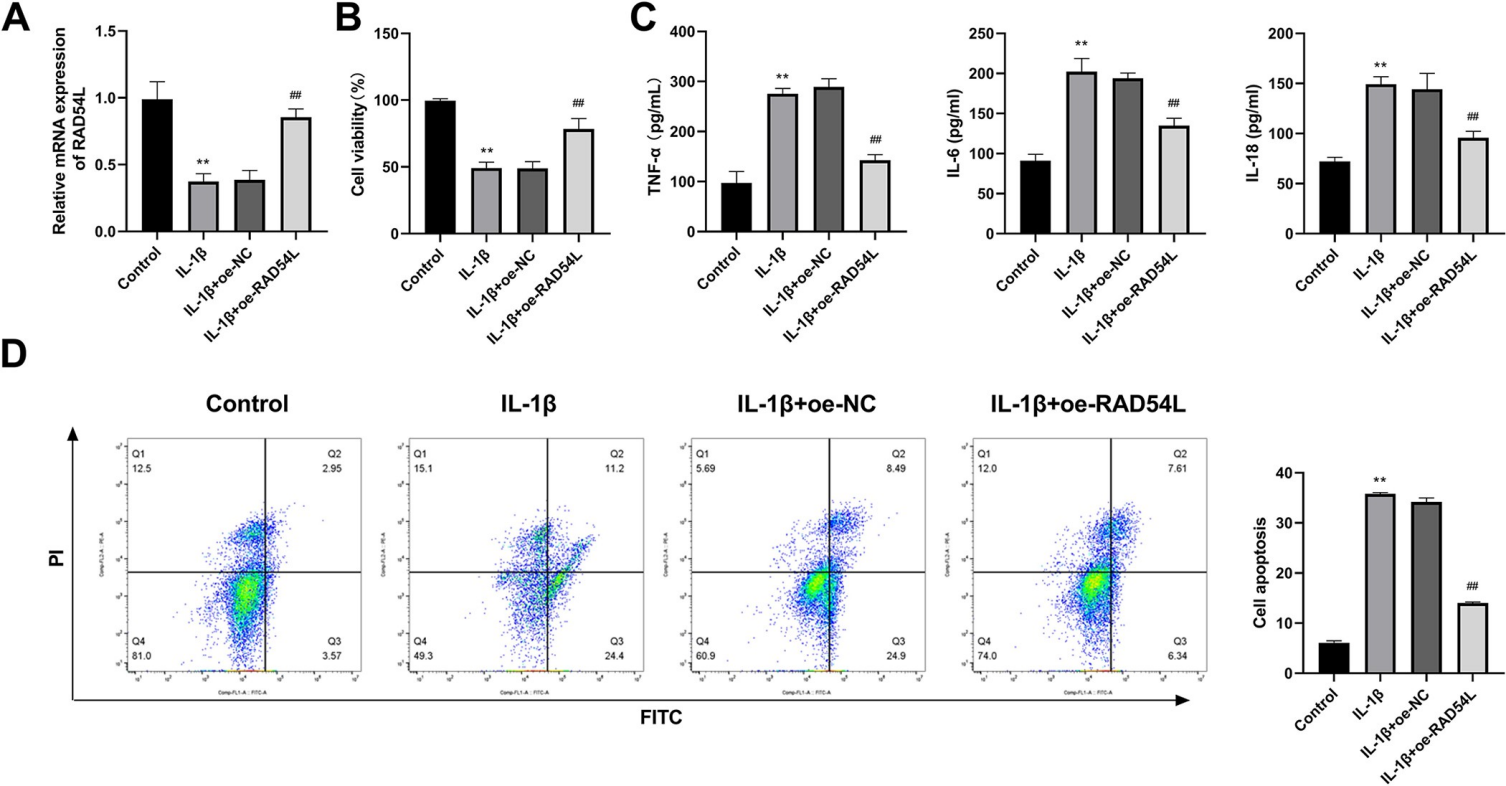
RAD54L overexpression promotes cell viability and inhibits the inflammatory response and apoptosis of IL-1β-induced human chondrocytes. **(A)** RAD54L expression was detected by RT-qPCR. **(B)** Cell viability was detected by CCK-8 in chondrocytes. **(C)** The levels of proinflammatory cytokines (IL-6, IL-18, and TNF-α). **(D)** Flow cytometry was used to detect the apoptotic rates of chondrocytes. **P < 0.01 *vs*. the Control group. ^##^ P < 0.01 *vs*. the IL-1β group.

Based on the findings of previous studies of each gene, RAD54L was selected for further research. IL-1β is reported to be related to growth inhibition and apoptosis induction in chondrocytes [36]. Therefore, we examined the effects of RAD54L on human chondrocytes with and without IL-1β induction. We found that RAD54L expression was significantly down-regulated in human chondrocytes induced by IL-1β (P < 0.01, Fig 6A). The CCK-8 assay results demonstrated that the viability of human chondrocytes was reduced in the IL-1β group, whereas the cell viability was significantly increased in the IL-1β+oe-RAD54L group (P < 0.01, Fig 6B). Decreased expression levels of IL-6, IL-18, and TNF-α were observed in the IL-1β+oe-RAD54L group, but increased expression levels of three cytokines were observed in the IL-1β group (P < 0.01, Fig 6C). Flow cytometry was used to validate the role of RAD54L in apoptosis. The results revealed that the apoptotic rate was increased in the IL-1β group compared with the Control group, but was significantly inhibited in the IL-1β+oe-RAD54L group (P < 0.01, Fig 6D).

### RAD54L overexpression attenuates OA in rats

**Fig 7:**
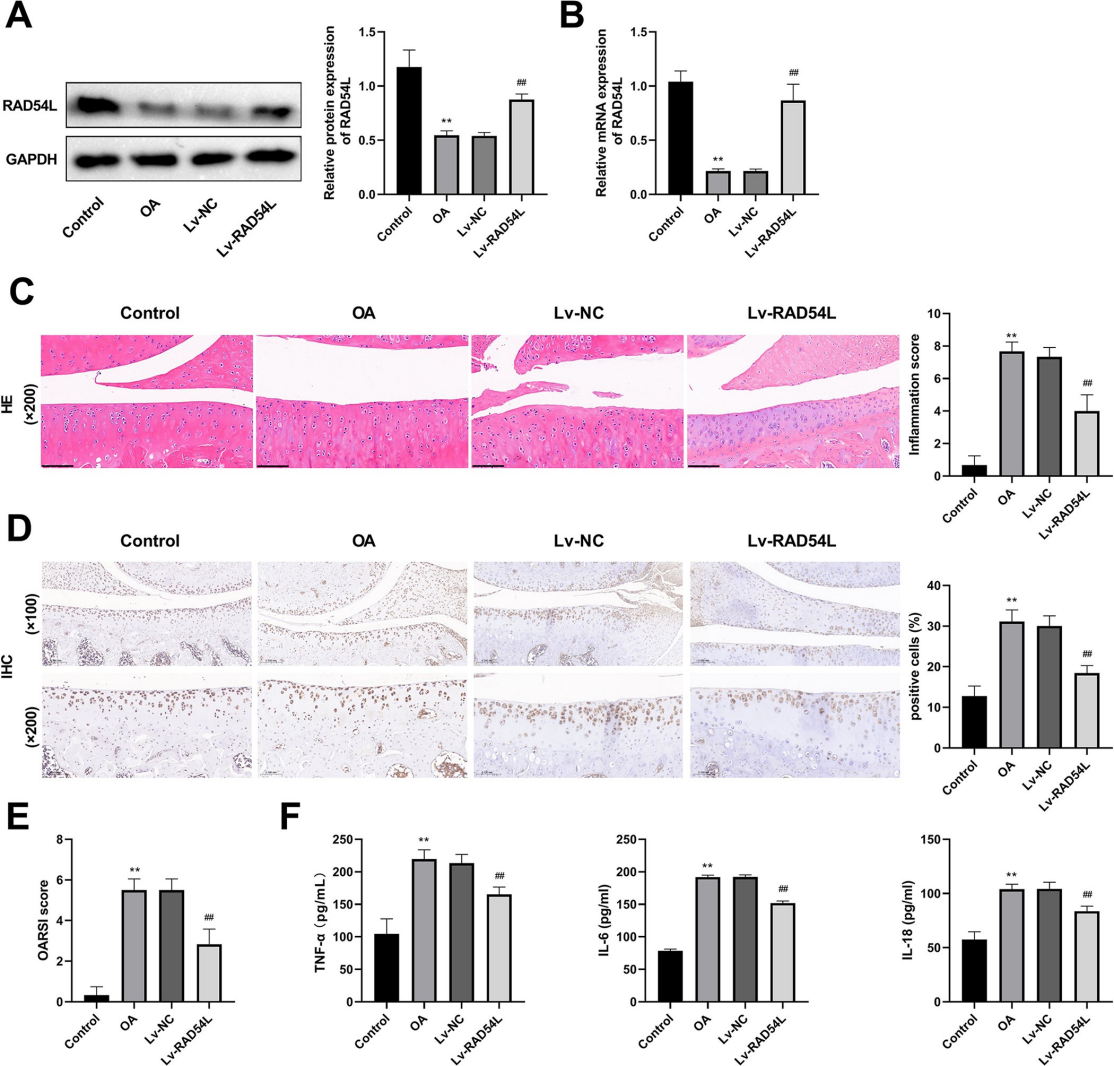
RAD54L overexpression attenuates osteoarthritis in rats. **(A)** The protein expression level of RAD54L. **(B)** The relative mRNA expression level of RAD54L. **(C)** The results of hematoxylin-eosin staining (200×, scale bars = 100 μm) and the inflammation score. **(D)** The immunohistochemical staining of cleaved-caspase-3 (100× or 200×, scale bars = 200 or 100 μm). **(E)** The Osteoarthritis Research Society International score. **(F)** The levels of proinflammatory cytokines (TNF-α, IL-6, and IL-18) in serum. ** P < 0.01 *vs*. the Control group. ^##^ P < 0.01 *vs*. the IL-1β group.

We further studied the role of RAD54L in rats with OA by overexpressing it. The results of Western Blot and RT-qPCR showed that the expression of RAD54L in the OA group was significantly decreased compared with that in the Control group (P < 0.01, Fig 7A and 7B). Compared with the OA group, RAD54L expression was significantly increased in the Lv-RAD54L group (P < 0.01, Fig 7A and 7B). The results of H&E staining showed that the arrangement of the cartilage growth plates of rats in the Lv-RAD54L group was more ordered than that in the OA group (Fig 7C), and the inflammation score decreased after the up-regulation of RAD54L (P < 0.01, Fig 7C). There was an obvious reduction in the number of cleaved-caspase-3 positive cells in the cartilage region in the Lv-RAD54L group compared to the OA group (P < 0.01, Fig 7D), indicating that the apoptosis level of chondrocytes was inhibited. Compared with the OA group, a lower OARSI score was found in the Lv-RAD54L group, suggesting a lower OA severity (P < 0.01, Fig 7E). Up-regulation of RAD54L suppressed the expression of TNF-α, IL-6 and IL-18 (all P < 0.01, Fig 7F), which were promoted by OA. The above results showed that overexpression of RAD54L was involved in improving the progression of OA in rats.

### Overexpression of RAD54L inhibits the HIF-1α/VEGF signaling pathway

**Fig 8:**
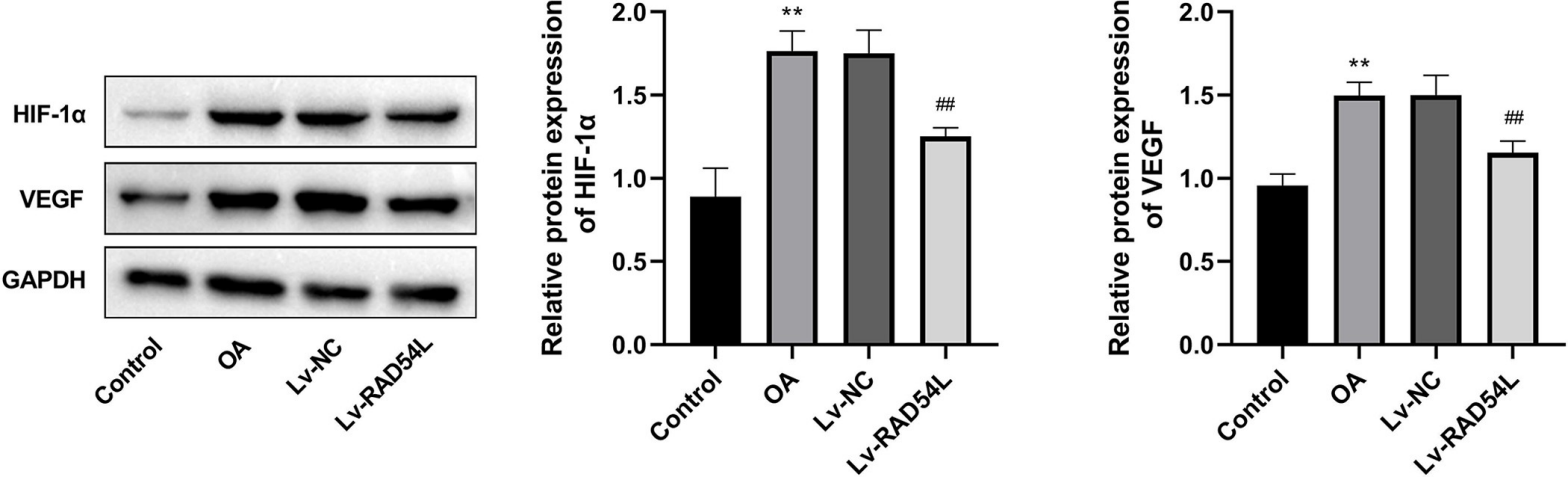
The relative protein expression of HIF-1α and VEGF. ** P < 0.01 *vs*. the Control group. ^##^ P < 0.01 *vs*. the IL-1β group.

To further elucidate the regulatory mechanism of RAD54L in OA, we predicted and verified the possible signaling pathway of RAD54L. There were 283 genes between OA-related genes and genes co-expressed with RAD54L (S4A Fig), which were subjected to KEGG enrichment analysis. Then we selected the HIF-1 signaling pathway for further validation based on our results and those of previous studies (S4B Fig). We examined the expression of HIF-1α, a key protein of the HIF-1 pathway, and VEGF, a known HIF-1 target gene, in the cartilage tissues of rats with OA. The HIF-1α/VEGF signaling pathway was stimulated in the OA group based on the elevated expression of HIF-1α and VEGF, while overexpression of RAD54L inhibited this signaling pathway by decreasing the relative expression level of HIF-1α and VEGF (all P<0.01, Fig 8).

## Discussion

OA is the leading cause of disability worldwide and has brought a substantial burden to society and a decreased quality of life to those affected [37, 38]. However, the early OA diagnosis of OA is difficult owing to a lack of a comprehensive understanding of the main molecular mechanisms of OA and effective biomarkers. Currently available bioinformatics methods present a wide range of possibilities for enhancing the diagnosis and treatment of many diseases, including OA [39, 40]. In this study, we screened out six hub genes by bioinformatics analysis. Then, we selected RAD54L for further analysis and found that RAD54L overexpression promoted cell viability, and inhibited the inflammatory response and apoptosis of IL-1β-induced human chondrocytes. In addition, overexpressing RAD54L in rats with OA contributed to a lower level of inflammation, a reduction in the apoptosis of chondrocytes, and an alleviation of the pathological changes in knee articular cartilage tissue, by suppressing the HIF-1α/VEGF signaling pathway.

An increasing number of genes and pathways have been found to be associated with OA by bioinformatics analysis. A study using single-cell RNA sequencing reveals three key genes related to the proliferation and differentiation of chondrocytes in OA, namely CD44, JUN, and Fibronectin-1 [41]. Zhang et al have identified 174 DEGs after bioinformatic analysis and found that the Forkhead box O signaling pathway and the IL-17 signaling pathway take part in the progression of OA [42]. Fibroblast activating protein and Zinc finger E-box binding homeobox 1 are screened by single-cell level analysis, which are the main regulators of the promotion of OA in cartilage and meniscus [43]. Yang et al have suggested activating transcription factor 3 as a promising diagnostic biomarker of early-stage OA [44]. Our work identified 81 co-DEGs by comparing genes expressed in OA samples compared to healthy subjects and finally revealed six hub genes, including RAD54L, HJURP, UBE2C, CCNB2, SMC4, and CENPN.

The loss of chondrocyte vitality and abnormal apoptosis are important hallmarks of human OA [45, 46]. Inflammatory mediators could promote the initiation and perpetuation of the OA process [47]. The low level of inflammation in OA is considered to be a major pathogenic factor for joint injury and joint pain [48, 49]. Therefore, increasing genes have shown potential value in the treatment of OA with the ability to regulate chondrocyte vitality, apoptosis, and inflammation. For example, down-regulation of receptor‑interacting protein kinase 4 has been reported to stimulate the proliferation and suppress the apoptosis of chondrocytes, thus playing a role in attenuating OA [50]. Sirtuin 3 overexpression ameliorates OA of rats by inhibiting inflammation and apoptosis of chondrocytes [51]. Silencing angiopoietin-like protein 4 can attenuate knee OA of mice due to the reduction of TNF-α-induced chondrocyte inflammation and apoptosis [52]. The up-regulation of fat-mass and obesity-associated gene can enhance cell viability, decrease the apoptotic rate, and inhibit the expression of inflammatory markers in lipopolysaccharide-induced normal human chondrocytes, demonstrating their potential as targets of OA therapy [53]. Similarly, our results showed that the upregulation of RAD54L stimulated chondrocyte viability and suppressed inflammatory response and apoptosis levels of chondrocytes *in vitro and vivo*, suggesting that RAD54L could play a protective role in OA.

HIF-1α plays an important role in the progression of OA. An obvious higher expression level of HIF-1α has been found in patients with more severe knee OA [54]. HIF-1α may promote cartilage degeneration through suppressing the expression of B-cell lymphoma-2 [55]. Increased expression levels of HIF-1α promote the pyroptosis of synoviocytes, leading to severe synovial fibrosis in knee OA [56]. VEGF is a target of HIF-1α, and an increase of HIF-1α levels promotes VEGF expression [57]. Increased VEGF expression levels are associated with OA progression including cartilage degeneration and pain [58, 59]. In the later stage of OA, VEGF levels are up-regulated in articular cartilage [60] and serum [61]. The VEGF-A signaling pathway has been observed to be activated in the articular cartilage of mice with knee OA [62]. The suppression of VEGF in chondrocytes can prevent the progression of OA [63]. In this study, overexpression of RAD54L decreased the relative expression level of HIF-1α and VEGF, indicating that RAD54L attenuated OA by restraining the HIF-1/VEGF signaling pathway.

## Conclusion

In summary, RAD54L was finally screened by bioinformatic analysis. Overexpression of RAD54L could inhibit inflammatory responses and cell apoptosis in IL-1β-induced human chondrocytes, and promote cell viability. Additionally, overexpression of RAD54L showed a protective effect on knee articular cartilage injury and suppressed inflammatory responses in OA rats, with the suppression of the HIF-1α/VEGF signaling pathway. Our results suggested RAD54L as a promising biomarker for OA, providing a new target for the treatment of OA.

## Supporting information

S1 Fig The Veen diagram of co-DEGs among GSE98918 and GSE51588. (TIF)

S2 Fig The protein-protein interaction (PPI) network and hub gene analysis of co-DEGs. (A) The PPI network of co-DEGs. (B) Hub genes in the PPI network.
(TIF)

S3 Fig Validation of the diagnostic value of Hub genes. **(A)** and **(B)** Receiver operating characteristic curves of Hub genes in GSE98918 and GSE51588 respectively.
(TIF)

S4 Fig The prediction for the possible signaling pathway of RAD54L. **(A)** A Venn diagram of co-expression genes. **(B)** A bubble plot of Kyoto Encyclopedia of Genes and Genomes enrichment analysis.
(TIF)

S1 Table Primer sequences for RT-qPCR. (DOC)

S1 Raw images (PDF)
