# Supplementary figures and images for: Overexpression of RAD54L attenuates osteoarthritis by suppressing the HIF-1α/VEGF signaling pathway: Bioinformatics analysis and experimental validation
Source: PLoS One. 2024 Apr 9;19(4):e0298575. doi: 10.1371/journal.pone.0298575 (PMC11003635; doi:10.1371/journal.pone.0298575)

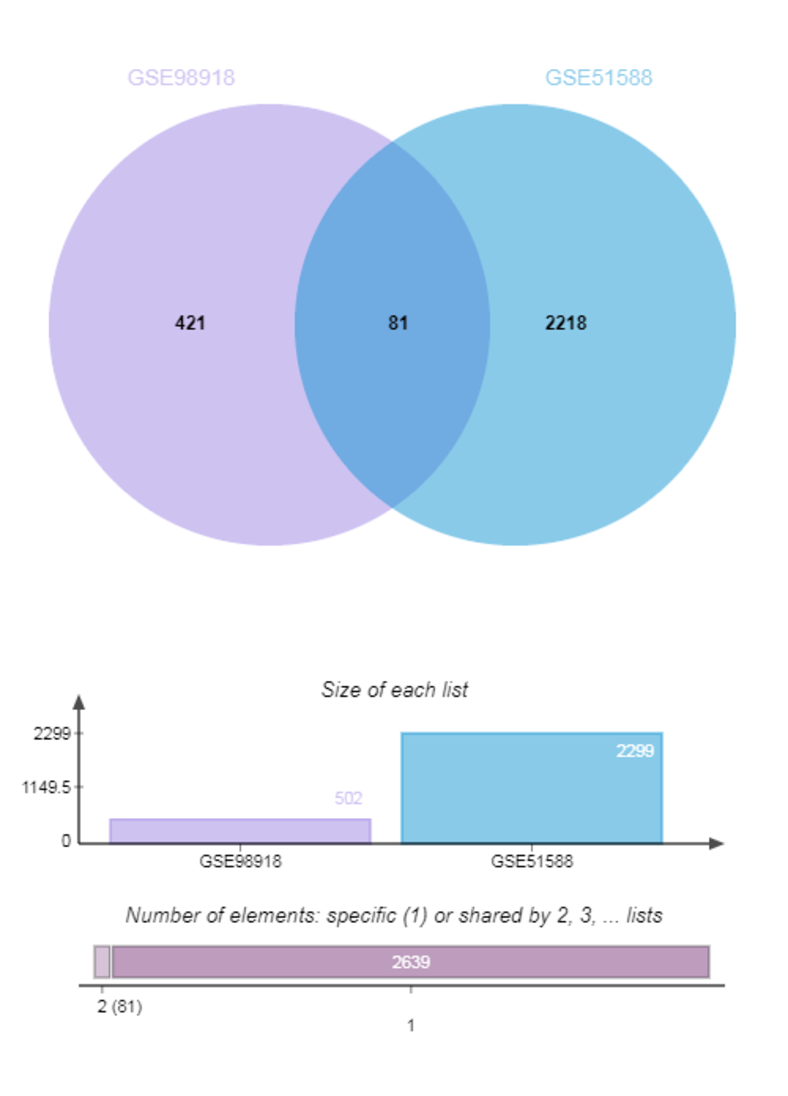

Supplement: S1 Fig — (TIF) [file pone.0298575.s001.tif]

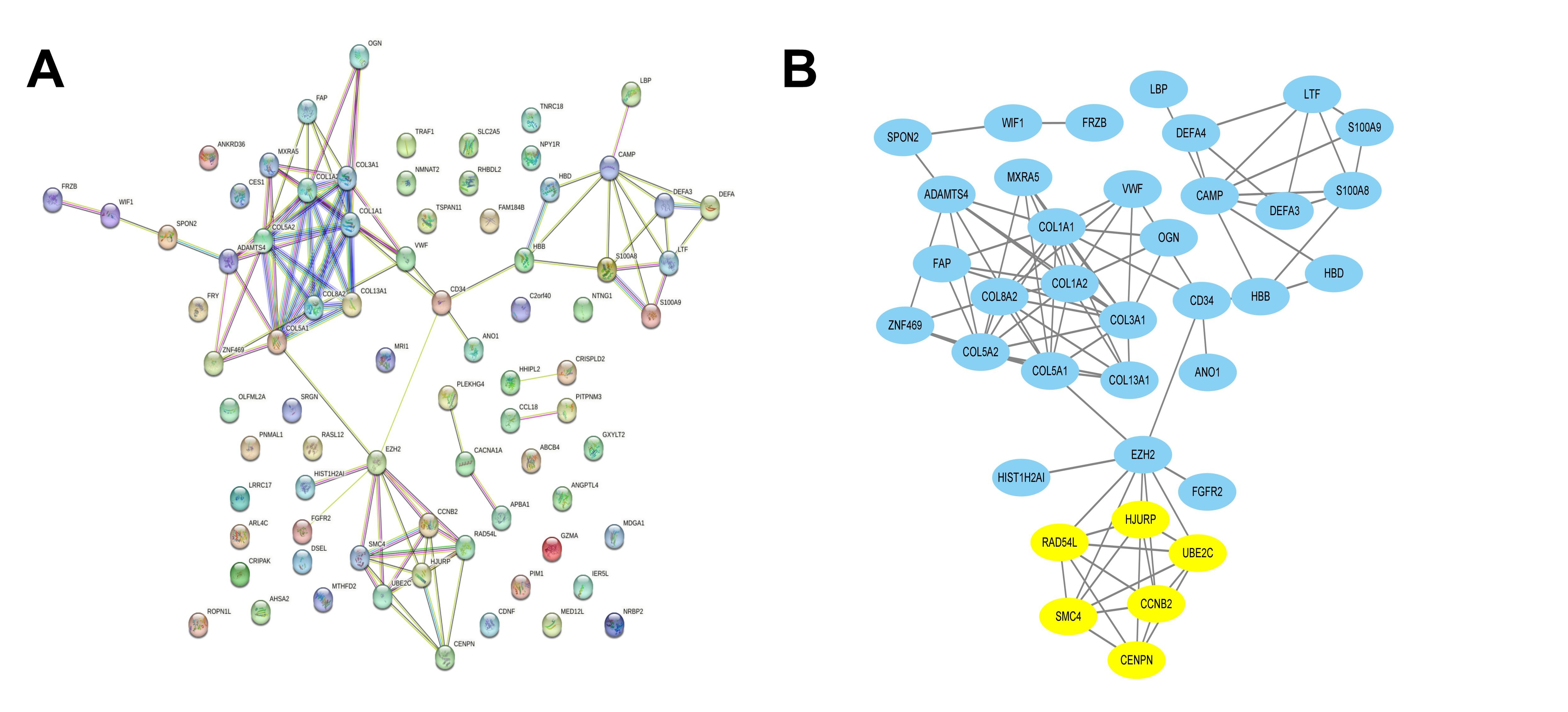

Supplement: S2 Fig — (A) The PPI network of co-DEGs. (B) Hub genes in the PPI network. (TIF) [file pone.0298575.s002.tif]

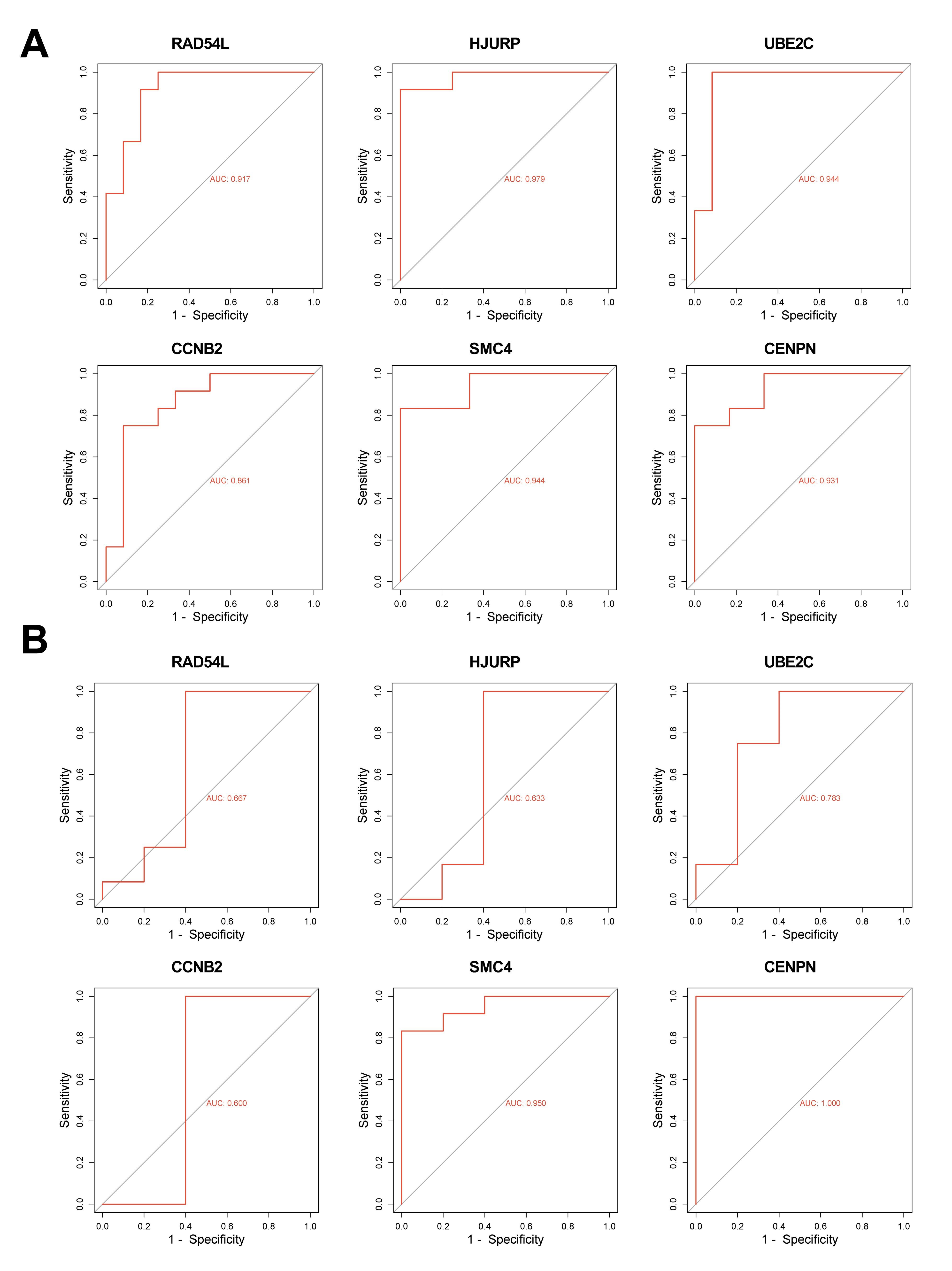

Supplement: S3 Fig — (A) and (B) Receiver operating characteristic curves of Hub genes in GSE98918 and GSE51588 respectively. (TIF) [file pone.0298575.s003.tif]

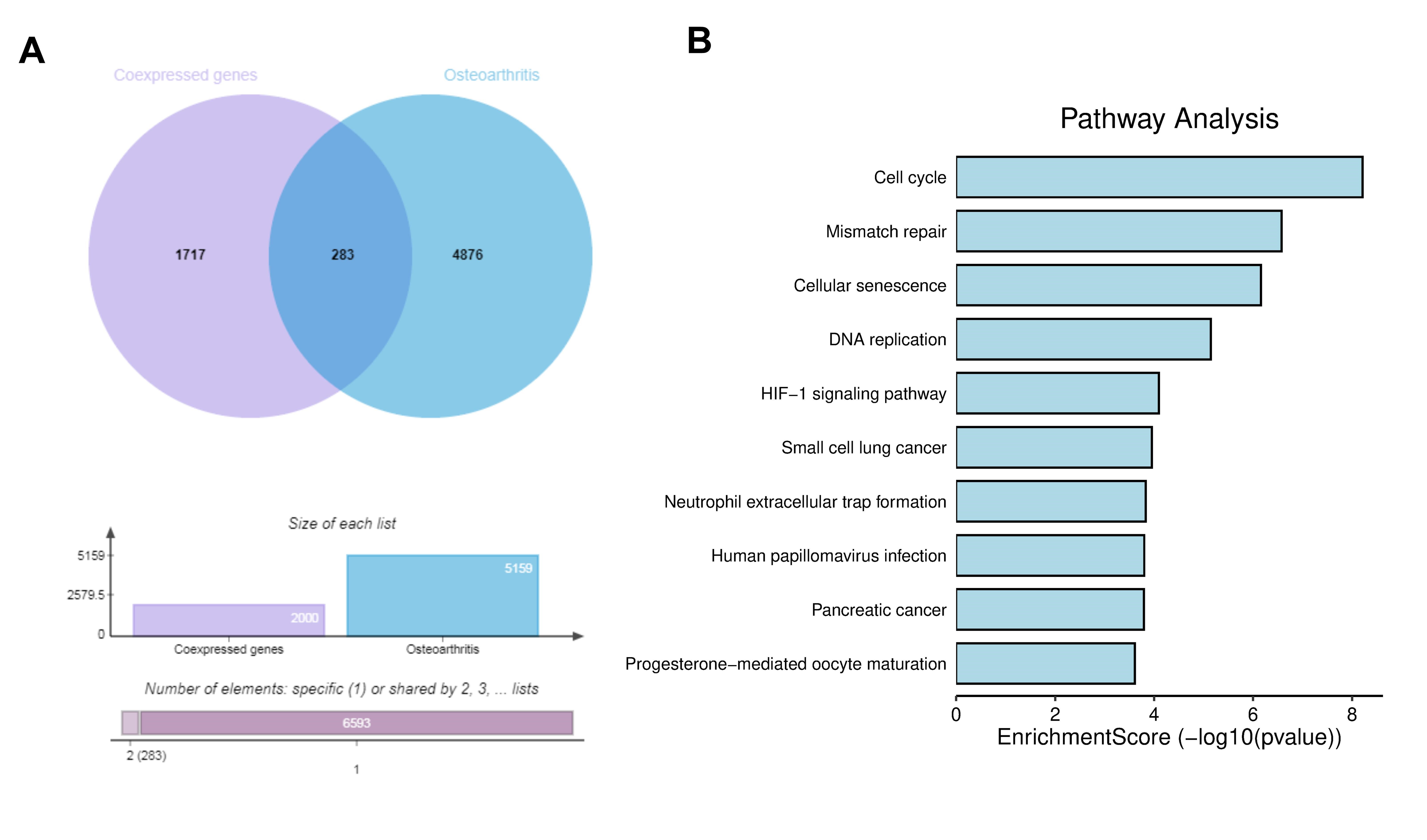

Supplement: S4 Fig — (A) A Venn diagram of co-expression genes. (B) A bubble plot of Kyoto Encyclopedia of Genes and Genomes enrichment analysis. (TIF) [file pone.0298575.s004.tif]

Fig.7A-RAD54L

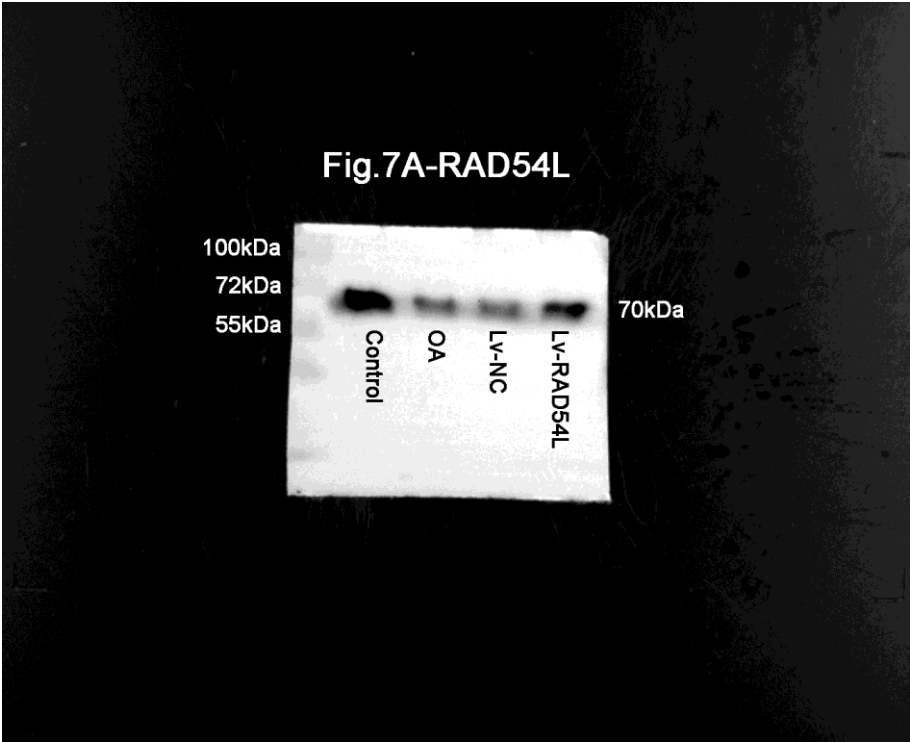

Fig.7A-GAPDH

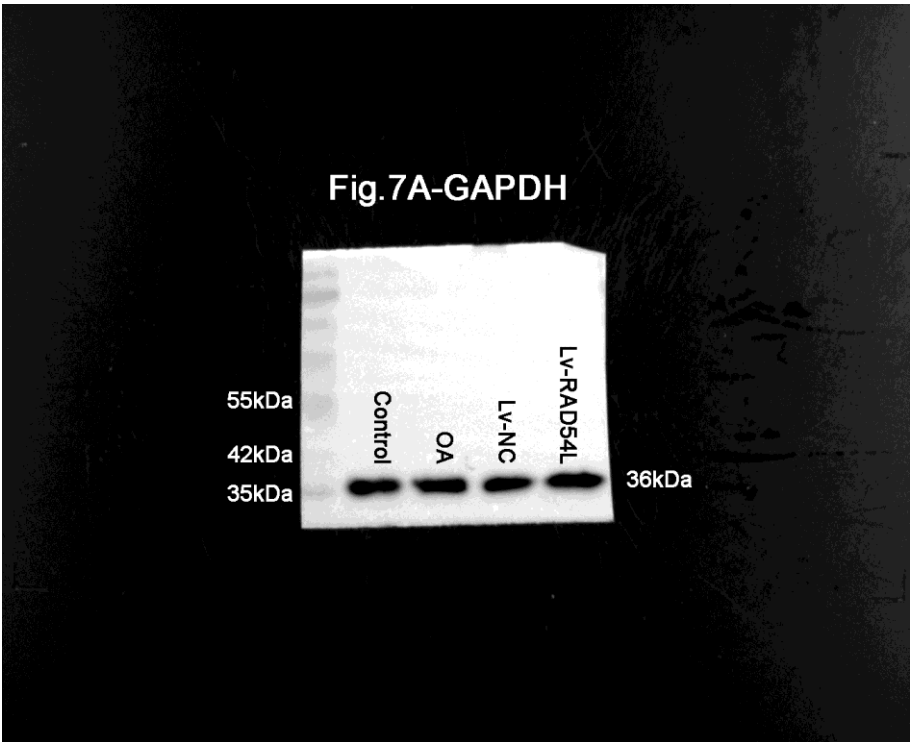

**Fig.8-HIF-1 $\alpha$**

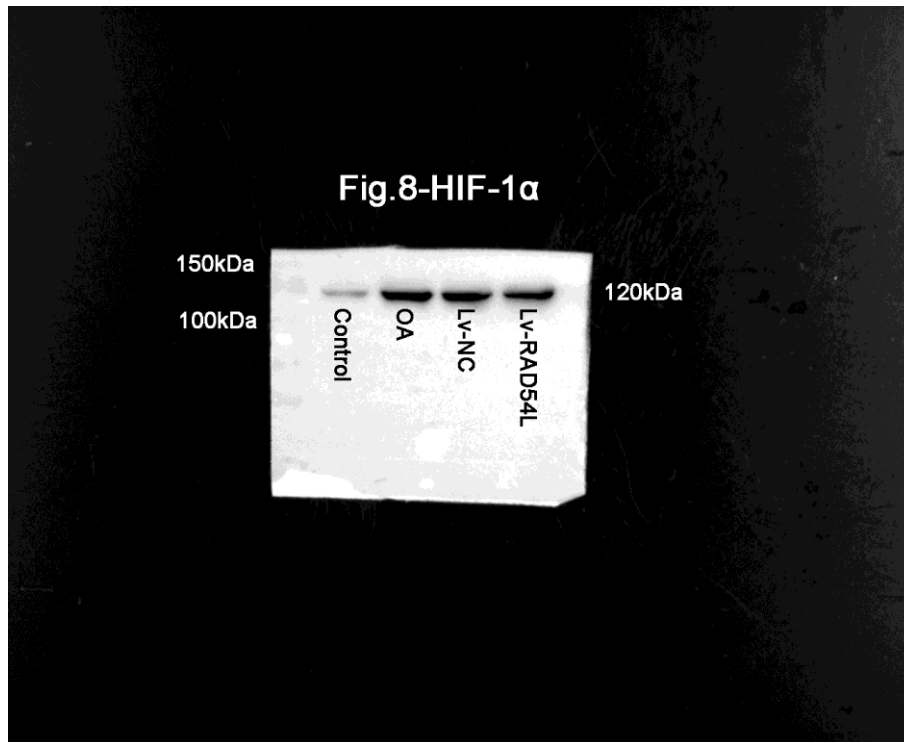

**Fig.8-VEGF**

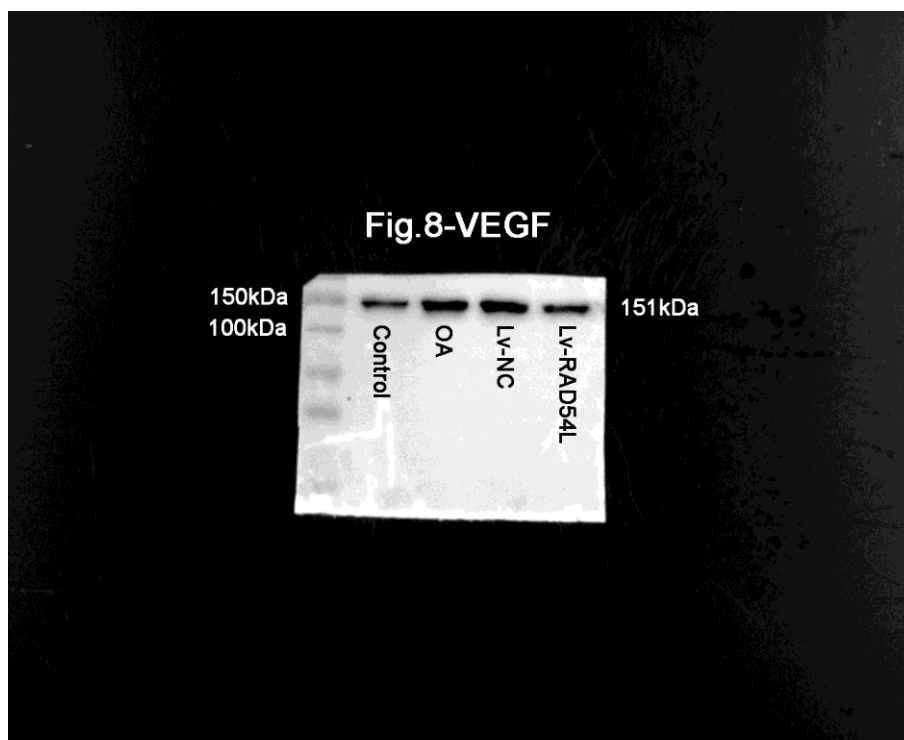

Fig.8-GAPDH

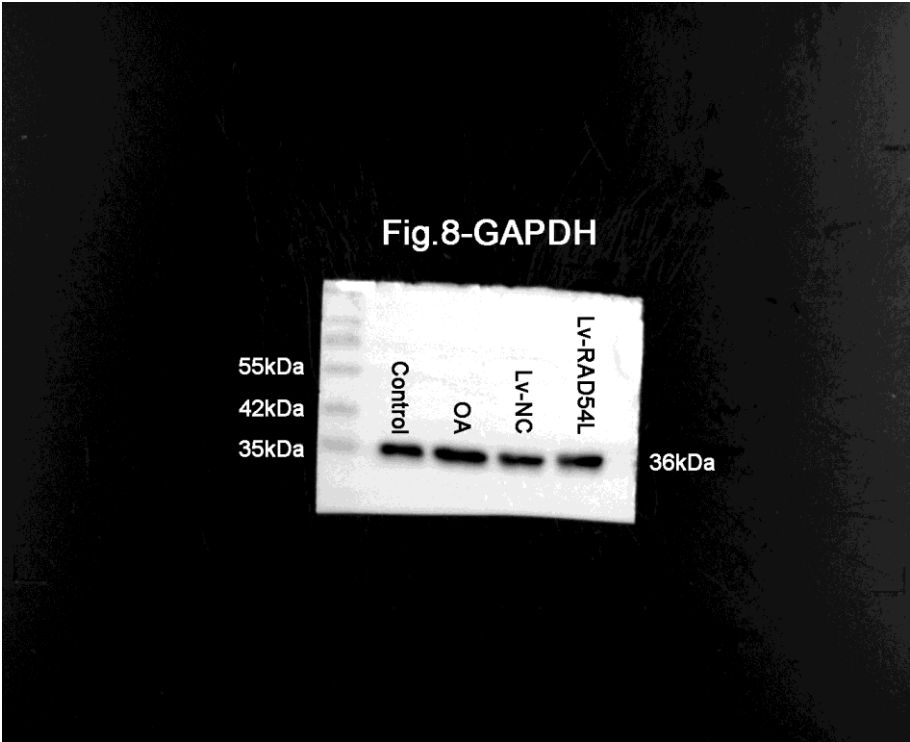

Supplement: S1 Raw images — (PDF) [file pone.0298575.s006.pdf]
