# Supplementary material for: Overexpression of RAD54L attenuates osteoarthritis by suppressing the HIF-1α/VEGF signaling pathway: Bioinformatics analysis and experimental validation
Source: PLoS One. 2024 Apr 9;19(4):e0298575. doi: 10.1371/journal.pone.0298575 (PMC11003635; doi:10.1371/journal.pone.0298575)
Supplement: S1 Table — (DOC) [file pone.0298575.s005.doc]

S1 Table. Primer sequences for RT-qPCR

| Name | Sequences（5’-3’） |
| --- | --- |
| GAPDH-F | CTCATGACCACAGTCCATGC |
| GAPDH-R | TTCAGCTCTGGGATGACCTT |
| SMC4-F | CCAGGGCTTATCAGAGCAAG |
| SMC4-R | CTTTCTCAGCCACAGCATCA |
| RAD54L-F | CTAACCGGCTGGTCATGTTT |
| RAD54L-R | AGCTGCTCAGAGCCTTCTTG |
| UBE2C -F | AAAAGGAGCAGAACCCTGCG |
| UBE2C -R | TGGGTGCGTTGTAAGGGTAG |
| HJURP-F | ATCCAGACTCCCCACAACAG |
| HJURP-F | AGCCTGATGCTCCTGAGGTA |
| CCNB2-F | CCGACGGTGTCCAGTGATTT |
| CCNB2-R | GCCGGTCTGGCTCTAACTTT |
| CENPN -F | CGTGAAGGAAGCCTTGGACT |
| CENPN -R | TGTGCGAACTCTAGCTGTGG |
